# Characterization of a Maltase from an Early-Diverged Non-Conventional Yeast *Blastobotrys adeninivorans*

**DOI:** 10.3390/ijms21010297

**Published:** 2019-12-31

**Authors:** Triinu Visnapuu, Aivar Meldre, Kristina Põšnograjeva, Katrin Viigand, Karin Ernits, Tiina Alamäe

**Affiliations:** Department of Genetics, Institute of Molecular and Cell Biology, University of Tartu, Riia 23, 51010 Tartu, Estonia; triinu.visnapuu@ut.ee (T.V.); aivarmeldre@gmail.com (A.M.); kristina.poshnograjeva@gmail.com (K.P.); katrin.viigand@gmail.com (K.V.); karin.ernits@gmail.com (K.E.)

**Keywords:** *Arxula adeninivorans*, glycoside hydrolase, α-glucosidase, maltose, panose, amylopectin, glycogen, inhibition by Tris, transglycosylation

## Abstract

Genome of an early-diverged yeast *Blastobotrys* (*Arxula*) *adeninivorans* (*Ba*) encodes 88 glycoside hydrolases (GHs) including two α-glucosidases of GH13 family. One of those, the *rna_ARAD1D20130g*-encoded protein (*Ba*AG2; 581 aa) was overexpressed in *Escherichia coli*, purified and characterized. We showed that maltose, other maltose-like substrates (maltulose, turanose, maltotriose, melezitose, malto-oligosaccharides of DP 4‒7) and sucrose were hydrolyzed by *Ba*AG2, whereas isomaltose and isomaltose-like substrates (palatinose, α-methylglucoside) were not, confirming that *Ba*AG2 is a maltase. *Ba*AG2 was competitively inhibited by a diabetes drug acarbose (K_i_ = 0.8 µM) and Tris (K_i_ = 70.5 µM). *Ba*AG2 was competitively inhibited also by isomaltose-like sugars and a hydrolysis product—glucose. At high maltose concentrations, *Ba*AG2 exhibited transglycosylating ability producing potentially prebiotic di- and trisaccharides. Atypically for yeast maltases, a low but clearly recordable exo-hydrolytic activity on amylose, amylopectin and glycogen was detected. *Saccharomyces cerevisiae* maltase MAL62, studied for comparison, had only minimal ability to hydrolyze these polymers, and its transglycosylating activity was about three times lower compared to *Ba*AG2. Sequence identity of *Ba*AG2 with other maltases was only moderate being the highest (51%) with the maltase MalT of *Aspergillus oryzae*.

## 1. Introduction

A non-conventional yeast *Blastobotrys adeninivorans* (syn. *Arxula adeninivorans*) belongs to a basal clade of Saccharomycotina subphylum and diverged in the evolution of fungi long before *Saccharomyces* [1,2,3,4,5]. A recent study states that the divergence of basal Saccharomycotina from *Saccharomyces cerevisiae* took place between 200 and 400 million years ago [4].

*B. adeninivorans* has several biotechnologically relevant properties: accumulation of lipids [6], salt tolerance, temperature-induced filamentation that promotes protein secretion and the ability to use a wide range of carbon and nitrogen sources, including purines, tannin and butanol, that are unusual nutrients for yeasts [2,7]. *B. adeninivorans* has been engineered for butanol production, applied in kits for the detection of hormones and dioxine in water as well as for manufacturing of tannase and cutinases [7]. Some other enzymes of *B. adeninivorans* have also been investigated, including alcohol dehydrogenase [8], extracellular glucoamylase [9] and invertase [10]. A highly active endo-inulinase of *B. adeninivorans* was cloned and recently characterized [11]. The genome of *B. adeninivorans* was sequenced in 2014 [2].

The genes potentially encoding α-glucosidases in the genomes of non-conventional yeasts were analysed in Viigand et al. [12]. The genes encoding two putative α-glucosidases designated as AG1 and AG2 were revealed in genome of *B. adeninivorans.* In the genomes of most yeasts addressed in Viigand et al. [12], the α-glucosidase genes resided in maltose utilization (*MAL*) clusters, whereas no *MAL* clusters were detected in *B. adeninivorans*.

α-Glucosidases have been popular objects to study protein evolution and phylogenesis [13,14,15,16], but they also have a biotechnological value. Thus, some of them have a high transglycosylating activity thanks to which they produce prebiotic oligosaccharides and potential functional food ingredients such as panose, melezitose, isomelezitose and isomalto-oligosaccharides [17,18,19,20,21]. For example, the α-glucosidase of *S. cerevisiae* produced isomelezitose from sucrose when the substrate concentration was high [21]. Transglycosylating ability of maltose by the α-glucosidase of *Xanthophyllomyces dendrorhous* (syn. *Phaffia rhodozyma*) has been studied in detail, and synthesis of tri- and tetrasaccharides with α-1,6 linkages was detected. This enzyme certainly has a biotechnological potential—it produced 3.6 times more transglycosylation products than the *S. cerevisiae* α-glucosidase studied at the same conditions [17,20].

Considering α-glucosidases of yeasts, they have mostly been studied in *S. cerevisiae* as these enzymes are crucial in baking and brewing [22]. *S. cerevisiae* has two types of α-glucosidases—maltases (EC 3.2.1.20) and isomaltases (EC 3.2.1.10)—that differ for substrate specificity. Maltases degrade maltose and maltose-like sugars, i.e., maltotriose, turanose and maltulose, but cannot degrade isomaltose and isomaltose-like sugars (α-1,6 linkages) such as palatinose. Both types of enzymes hydrolyze sucrose and a synthetic substrate *p*-nitrophenyl-α-d-glucopyranoside (*p*NPG) [15,16,23,24]. At the same time, some yeasts such as *Ogataea polymorpha* and *Scheffersomyces stipitis*, have promiscuous maltase-isomaltases that hydrolyze maltose- and isomaltose-like substrates [12,16,25].

In the current work, we expressed heterologously in *Escherichia coli* and biochemically characterized the α-glucosidase *Ba*AG2 of *B. adeninivorans* encoded by *rna_ARAD1D20130g*. We confirmed that *Ba*AG2 is a maltase with a considerable transglycosylating activity. Not typical for yeast maltases, *Ba*AG2 had exo-hydrolytic activity on amylose, amylopectin and glycogen. *Ba*AG2 is the first maltase characterized from *B. adeninivorans.*

## 2. Results

### 2.1. In Silico Analysis of BaAG2

According to annotations provided at the MycoCosm website [26], the genome of *Blastobotrys* (*Arxula*) *adeninivorans* [2] encodes 185 carbohydrate-active enzymes, including 88 glycoside hydrolases (GHs) assigned to different families. When mining the genome of *B. adeninivorans* [2] for the genes related to maltose hydrolysis, we found two genes encoding intracellular GH13 family proteins. Respective proteins were designated as AG1 and AG2 [12]. In MycoCosm, the AG1 was annotated as a protein similar to maltase Mal1 of *Schizosaccharomyces pombe* and the AG2 as similar to maltases of filamentous fungi *Aspergillus* and *Penicillium*. Both of these proteins were predicted to lack a signal peptide and to locate intracellularly. We confirmed this by using the SignalP program (see Materials and Methods). Aside from these two GH13 proteins, three putative extracellular α-glucosidases of GH31 family were detected in *B. adeninivorans* genome (see Table S1 of Supplementary Materials). Table S1 also includes two *B. adeninivorans* enzymes that have been experimentally studied: a secreted invertase AINV belonging to GH31 family [10] and a secreted glucoamylase [9] of GH15 family.

**Figure 1:**
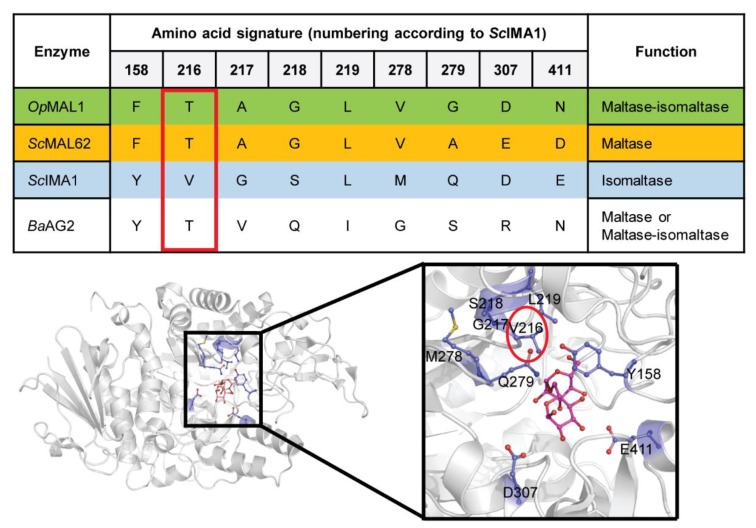
Amino acid signature of yeast α-glucosidases, including *B. adeninivorans* AG2 (upper panel) and their designation on the three-dimensional (3D) structure of *S. cerevisiae* isomaltase IMA1 in complex with isomaltose (RCSB Protein Data Bank, PDB: 3AXH [29]) (lower panel). Location of Val216 in the structure is marked with a red circle.

Substrate specificity of α-glucosidases can be *in silico* predicted based on so-called amino acid signature—a set of amino acids that locate in the vicinity of the substrate-binding pocket [12,15,27,28]. The upper panel of Figure 1 shows the amino acid signature of *O. polymorpha* maltase-isomaltase MAL1, *S. cerevisiae* maltase MAL62, isomaltase IMA1, and *B. adeninivorans* AG2. The amino acids of these proteins corresponding to Val216 of *Sc*IMA1 are shown inside a red frame as this position is considered of key importance in selective substrate binding [28].

We then visualized the *S. cerevisiae* IMA1 structure in complex with isomaltose (PDB: 3AXH) [29] using PyMol [30] in order to display all these amino acids (Figure 1, lower panel). In *Ba*AG2, a Thr is present at position of Val216 and therefore we predicted that this enzyme is most probably a maltase. However, as maltase-isomaltases also have a Thr at that position (Figure 1, upper panel; [12,16]), based on the amino acid signature, *Ba*AG2 may also be a promiscuous enzyme with a wide substrate spectrum like *O. polymorpha* MAL1.

**Figure 2:**
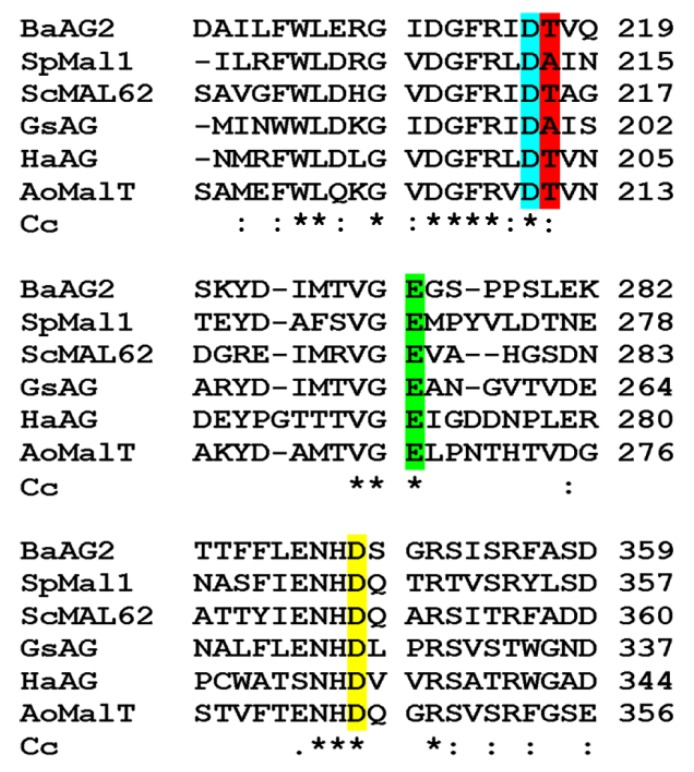
Fragments of sequence alignment of six maltases. *Ba*AG2, *Blastobotrys adeninivorans* AG2 (580 aa); *Sp*Mal1, *Schizosaccharomyces pombe* Mal1 (579 aa, NP_595063.1) [33]; *Sc*MAL62, *Saccharomyces cerevisiae* MAL62 (584 aa, P07265) [23]; *Gs*AG, *Geobacillus stearothermophilus* exo-α-1,4-glucosidase (555 aa, BAA12704.1) [34]; *Ha*AG, *Halomonas* sp. H11 α-glucosidase (538 aa, BAL49684.1) [35]; *Ao*MalT, *Aspergillus oryzae* maltase MalT (574 aa, XP_001825184.1) [36]. Highlights: catalytic nucleophile (turquoise), acid-base catalyst (green), a transition state stabilizer (yellow) and a residue crucial for substrate specificity (red). Cc, Clustal consensus. Marking below the sequence alignment is according to Clustal consensus showing conservation: * positions with fully conserved residue; : positions with residues of strongly similar properties; . positions with residues of weakly similar properties.

Figure 2 presents fragments of sequence comparison of *Ba*AG2 with those of five experimentally studied maltases from GH13 family: two from bacteria, two from yeasts and one from a filamentous fungus *Aspergillus*. The identity matrix of these proteins is presented in Supplementary Materials (Table S2). Though the proteins aligned sufficiently well over the entire sequence, *Ba*AG2 showed only moderate sequence identity with the other maltases ranging from 35% with *Halomonas* sp. H11 α-glucosidase to 51% with *Aspergillus oryzae* maltase MalT (Table S2). *In silico* assay of α-glucosidases of non-conventional yeasts [12] identified a putative α-glucosidase protein AG1 of *Lipomyces starkeyi* as the closest homologue (50% identity) of *Ba*AG2. The amino acid signature of the *Lipomyces* protein was YTVNKLSHE, and it was, therefore, predicted as a maltase [12].

The GH13 enzymes use an Asp (D) as a nucleophile and a Glu (E) as an acid-base catalyst [31]. Additionally, an Asp of the conserved ‘NHD’ motif serves as a transition state stabilizer [32]. In the *Ba*AG2 protein, Asp216 was predicted as a nucleophile, Glu274 as an acid-base catalyst and Asp348 as a stabilizer (Figure 2). Thr217 is located next to the catalytic nucleophile Asp216 in *Ba*AG2 (Figure 2). In maltases and maltase-isomaltases, either Thr or Ala is present at respective position, whereas in isomaltases, a Val is present [12,15,27,28], indicating that a Val residue at this position interferes with hydrolysis of maltose-like substrates. Indeed, if respective Thr was substituted with Val in *O. polymorpha* maltase-isomaltase, utilization of maltose-like sugars was severely hampered [16]. Furthermore, after substitution of Val216 in *S. cerevisiae* IMA1 with Thr, the isomaltase IMA1 gained the ability to hydrolyze maltose [28,29].

### 2.2. Maltose-Like and Isomaltose-Like Sugars Are Growth Substrates for B. adeninivorans

**Figure 3:**
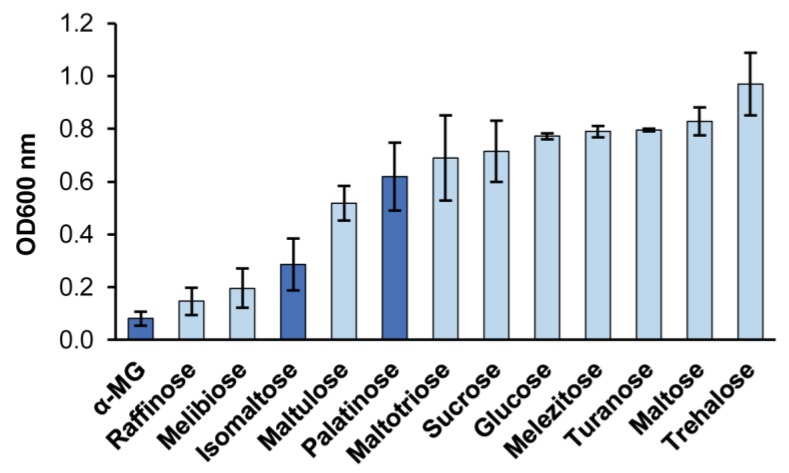
Growth of *B. adeninivorans* on sugars (supplemented at 2 g/L) evaluated by an optical density (OD) of the culture at 600 nm achieved by 24-h cultivation on a microplate at 37 °C. Isomaltose and isomaltose-like sugars are indicated by dark blue bars. Error bars are representing standard deviations (SD) of two independent experiments with two replicates.

According to the information present in the CBS-KNAW culture collection [37], *B. adeninivorans* CBS 8244 used in current work assimilates following α-glucosidic sugars: maltose, sucrose, melezitose, trehalose and α-MG. Of those, melezitose is a maltose-like sugar, and α-MG (a synthetic analogue of isomaltose [38]) is an isomaltose-like sugar. Glucose and many other monosaccharides are also assimilated. We asked if *B. adeninivorans* can also assimilate some other α-glucosidic sugars. We cultivated *B. adeninivorans* on Yeast Nitrogen Base (YNB) mineral medium containing 2 g/L of sugars indicated in Figure 3, and evaluated growth according to an optical density (OD) of 600 nm achieved by 24 h of growth (Figure 3). Our assay confirmed that five above-mentioned α-glucosidic sugars were indeed all assimilated. In addition, maltotriose, maltulose, turanose (maltose-like sugars) as well as isomaltose and palatinose (an isomaltose-like sugar) were identified as new growth substrates for this yeast. Thus, *B. adeninivorans* grows on both maltose-like and isomaltose-like sugars, meaning that it should possess enzymes for the hydrolysis of both types of sugars.

### 2.3. Cloning of the *Ba*AG2 Gene and Heterologous Expression of the *Ba*AG2 Enzyme

The *Ba*AG2 protein deduced from the gene is 580 aa long. The protein was predicted as intracellular—no secretion signal was detected by the SignalP program v. 5.0 [39]. *Ba*AG2 was overexpressed in *E. coli* with the His_6_-tag in its C-terminus that enabled purification of the protein using Ni^2+^-affinity chromatography. The calculated molecular weight of the protein was 67.9 kDa and a prominent band of respective size was detected by electrophoresis of the lysate produced from induced *E. coli* cells overexpressing the *AG2* gene (Figure S1). The *E. coli* lysate exhibited catalytic activity of 1 mM *p*NPG hydrolysis at 30 °C (71 U/mg), which after purification of the protein increased 5.8 times, reaching 411.5 U/mg.

### 2.4. Properties of *Ba*AG2

#### 2.4.1. Dependence of the *Ba*AG2 Activity on Temperature and pH. Thermal Stability of *Ba*AG2

The pH optimum of *Ba*AG2 was in moderately acidic region—from 5.5 to 6.5 (Figure S2). At pH 7.5, the activity was 81% from the maximum, and at pH 8.5, it was decreased to 52%. At pH 4.5 and 4.4, the respective values were 93 and 15%. Thus, the activity of *Ba*AG2 was significantly reduced at pH values below 4.5 and over 8.5 (Figure S2). The pH optimum of the *O. polymorpha* maltase is 6.0–6.5 [40], of *S. cerevisiae* maltase 6.5–6.8 [23,24] and of *Schizosaccharomyces pombe* maltase 6.0 [33]. In the current work, we routinely used the buffer with pH of 6.5 to characterize substrate specificity, kinetics and other properties of the enzyme.

**Figure 4:**
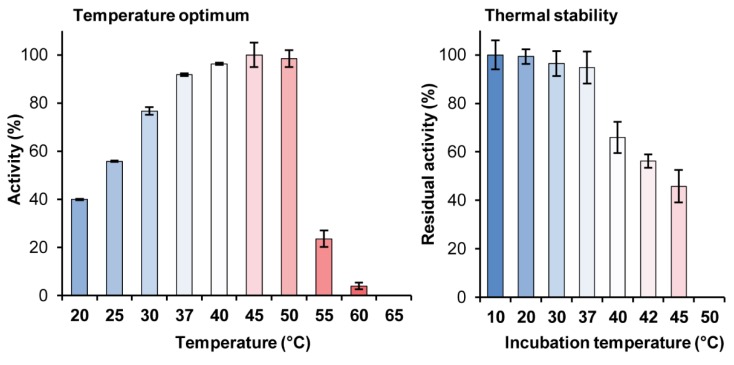
The effect of temperature on activity (left panel) and stability (right panel) of *Ba*AG2. For the thermostability assay, the enzyme was incubated for 30 min at the indicated temperature and residual activity was determined at 30 °C with *p*NPG as a substrate (see Materials and Methods, paragraph 4.4. for details). SDs of two to three replicates at each temperature are shown by error bars.

As shown in Figure 4 (left panel), catalytic activity of *Ba*AG2 was the highest at 45 °C being 24% higher than activity measured at 30 °C—the temperature we routinely used for enzyme activity assay. Figure 4 shows that at temperature over 50 °C, the activity of the enzyme rapidly declined. Thermal stability assay of *Ba*AG2 indicated that the enzyme was rather thermolabile: if kept for 30 min at temperatures above 37 °C, its catalytic activity reduced significantly (Figure 4, right panel). Therefore, we routinely performed enzymatic assays at 30 °C since some reactions (e.g., transglycosylation and polysaccharide degradation assays) were conducted up to several days.

#### 2.4.2. The Hydrolysis of Maltose and Maltose-Like Sugars

**Figure 5:**
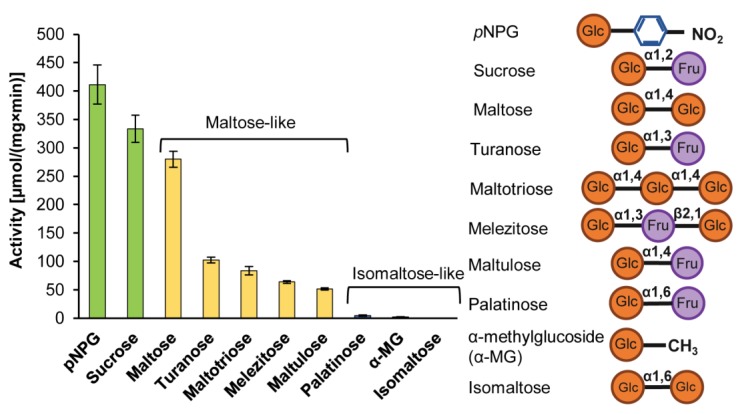
Activity of *Ba*AG2 on 100 mM sugars and 1 mM *p*NPG. Universal substrates are indicated by green, maltose and maltose-like sugars by yellow, and isomaltose and isomaltose-like sugars by blue bars. The composition and linkage types of the tested substrates are indicated. SD values of three to five replicates on each substrate are indicated.

We predicted *in silico* that *Ba*AG2 is either maltase or maltase-isomaltase (see Figure 1). To find out which of the predictions was correct, the purified *Ba*AG2 protein was reacted with a selection of 100 mM sugars and 1 mM *p*NPG that serves as a substrate for maltases, isomaltases and maltase-isomaltases (Figure 5). According to our assay, *Ba*AG2 could hydrolyze universal substrates (*p*NPG and sucrose), maltose and maltose-like sugars such as turanose, maltotriose, melezitose and maltulose. Isomaltose and isomaltose-like substrates palatinose and α-methylglucoside were not hydrolyzed (Figure 5). Therefore, *Ba*AG2 should be considered as a maltase.

#### 2.4.3. The Kinetic Parameters of Hydrolysis of Maltose, Maltose-Like and Universal Substrates

We studied the kinetics of the hydrolysis of *p*NPG, maltose, sucrose, maltotriose, melezitose, maltulose and turanose to calculate K_m_, V_max_, *k*_cat_ and catalytic efficiency (*k*_cat_/K_m_) values for these substrates. Results are presented in Table 1.

Table 1 shows that natural sugars, maltose and sucrose (α-d-Glc-(1→2)-β-d-Fru) were hydrolyzed by *Ba*AG2 with the highest catalytic efficiency (*k*_cat_/K_m_). Additionally, *Ba*AG2 had a high affinity and activity towards a synthetic substrate *p*NPG—the K_m_ for *p*NPG was 0.76 mM and the V_max_ over 750 U/mg. Affinity of *Ba*AG2 for maltose was slightly higher than for sucrose. From this aspect, *Ba*AG2 differs from the maltases of *S. cerevisiae* [23,24] and *Candida albicans* [41], and also from the maltase-isomaltase of *O. polymorpha* [16,40,42], for which affinity for sucrose is about twice higher than for maltose. In contrast, the maltase of *Schizosaccharomyces pombe* prefers maltose to sucrose [33]. Among the substrates tested, the affinity and catalytic efficiency of *Ba*AG2 was the lowest for melezitose. Thin layer chromatography (TLC) analysis showed that the glycosidic bond of turanose moiety of melezitose was hydrolyzed first, yielding sucrose and glucose as products (Figure S3). Similar mode of melezitose hydrolysis was earlier shown for the maltase-isomaltase of *O. polymorpha* [16]. Interestingly, isomelezitose was hydrolyzed by *Ba*AG2 with release of palatinose (Figure S3).

We would like to emphasize that the activity of *Ba*AG2 (Table 1) was higher compared to some of other studied maltases of yeasts and filamentous fungi. For example, its V_max_ on maltose was 7.5 times higher compared to *S. cerevisiae* maltase MAL62 [23], and over two times higher compared to maltase-isomaltase of *O. polymorpha* [16]. On the other hand, α-glucosidases of *X. dendrorhous* and *A. niveus* preferred polysaccharides such as starch, amylopectin and glycogen, and their *k*_cat_ values on maltose were respectively 25 and 28 times lower compared to the value of *Ba*AG2 [43,44]. Catalytic constant of α-glucosidase of *A. niger* on maltose was 144 1/s (2.6 times lower than *Ba*AG2), but the affinity towards maltose was very high (0.75 mM) [45]. Based on the literature, only one α-glucosidase of yeast and filamentous fungi has much higher *k*_cat_ on maltose than that of *Ba*AG2—the extracellular α-glucosidase of *Schizosaccharomyces pombe* (*k*_cat_ = 709 1/s) [46].

#### 2.4.4. The Inhibition Studies of Acarbose, Tris, Isomaltose-Like Sugars and Glucose

Having seen that isomaltose and isomaltose-like sugars are not hydrolyzed by *Ba*AG2 (Figure 5), we measured inhibition of *p*NPG hydrolysis reaction by these sugars as in [16]. Because maltases are usually strongly inhibited by glucose and acarbose, and much less by fructose [16], these substrates were also assayed as potential inhibitors of *Ba*AG2. When testing the effect of different buffers during the experiments, we noticed that the activity of *Ba*AG2 was lost in Tris-HCl buffer. More precise assay of the effect of tris(hydroxymethyl)aminomethane (Tris) on *Ba*AG2 revealed strong inhibition of the enzyme by this compound (Table 2). All tested compounds inhibited *p*NPG hydrolysis competitively, and the most powerful inhibitors of *Ba*AG2 were acarbose, Tris and glucose.

**Figure 6:**
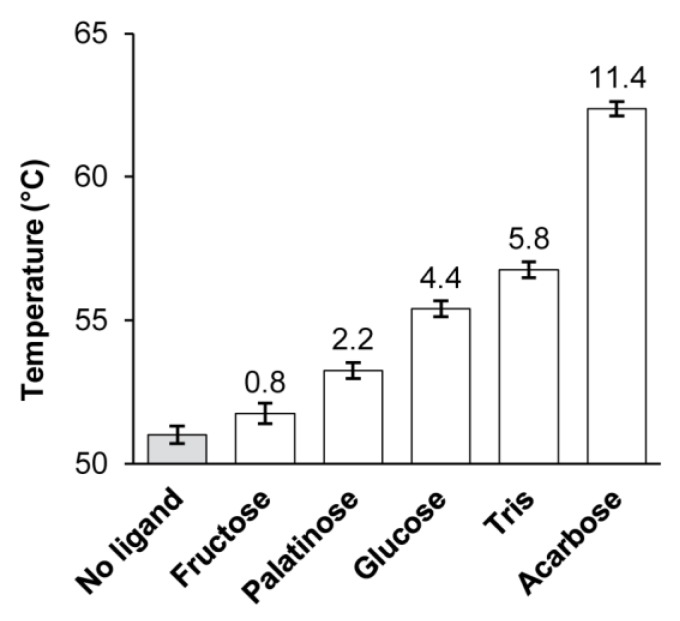
Thermostability of *Ba*AG2 in the presence and absence of indicated ligands. The T_m_ value of unliganded *Ba*AG2 (a grey bar) was 51 °C and the increase of T_m_ in the presence of a ligand is indicated above every bar. SDs of at least two independent experiments with two replicates at each condition are shown by error bars.

We expected that binding of the substrates or competitive inhibitors of the enzyme should increase its thermostability. To confirm this, we conducted a differential scanning fluorimetry (DSF) assay of *Ba*AG2 in the presence and absence of competing inhibitors as in [16,47]. Acarbose, palatinose, Tris and glucose (strong inhibitors of *p*NPG hydrolysis by *Ba*AG2) and fructose that inhibited the reaction only weakly (see Table 2) were used as ligands. The melting temperature (T_m_) values calculated from the DSF data are presented in Figure 6. The T_m_ of *Ba*AG2 without a ligand was similar to that of maltase-isomaltase MAL1 of *O. polymorpha*—51 °C [16]. Presence of acarbose (the strongest inhibitor of *Ba*AG2) increased the T_m_ of *Ba*AG2 by 11.4 °C. Presence of Tris raised the T_m_ value by 5.8 °C, and of glucose by 4.4 °C. Fructose and palatinose had only a minor effect on the T_m_ (Figure 6).

#### 2.4.5. The Hydrolysis of Malto-Oligosaccharides of DP 3 to 7

We have earlier shown that *O. polymorpha* maltase-isomaltase MAL1 could use maltotriose and maltotetraose as a substrate, while malto-oligosaccharides (MOS) of higher degree of polymerization (DP) were not hydrolyzed [16]. We showed that MOS of DP3 (see Table 1), DP4, DP5, DP6 and DP7 served as substrates for *Ba*AG2 (Figure S4). Assay of reaction course indicated that exo-hydrolysis occurred—a glucose residue was stripped off from the oligomer. Activity on MOS was only moderate and a substantial proportion of it stayed unreacted even after extended (22 h) reaction time (see Figure S4). The MAL62 of *S. cerevisiae* that was assayed alongside could not hydrolyze MOS longer than maltotetraose (DP4) (Figure S4).

#### 2.4.6. The Hydrolysis of Amylose, Amylopectin and Glycogen

Surprisingly, we detected the ability of *Ba*AG2 to hydrolyze polysaccharides that is a rather exceptional feature among maltases. After we noticed that *Ba*AG2 could hydrolyze soluble starch with the release of glucose, we performed a more detailed assay testing the hydrolysis of a set of polymeric α-glucans: amylose and amylopectin from potato, glycogen from oysters and dextrans of M_w_ 20 and 110 kDa. Commercial amylolytic enzymes amyloglycosidase (glucoamylase) from *Aspergillus niger,* and α-amylase from *Aspergillus oryzae* and *S. cerevisiae* MAL62 were used as reference enzymes.

**Figure 7:**
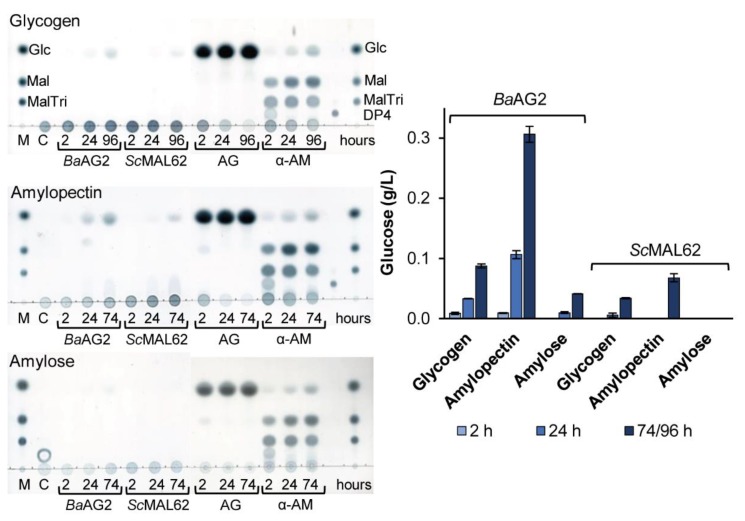
Hydrolysis of polysaccharides (5 g/L) by *Ba*AG2, *Sc*MAL62 and amyloglycosidase of *A. niger* (AG) and α-amylase of *A. oryzae* (α-AM). Samples withdrawn at indicated time points were analyzed using TLC. Reaction mixtures were spotted on TLC plates alongside with reference sugars (M): Glc (30 mM glucose); Mal (10 mM maltose); MalTri (10 mM maltotriose); DP4 (10 mM maltotetraose). The same marker sugars were used in all assays but are marked only on TLC plate of glycogen degradation. C—control sample without the enzyme but containing 5 g/L bovine serum albumin (BSA) incubated at the same conditions for 74/96 h. Glucose release was quantified enzymatically. See Materials and Methods, paragraphs 4.3 and 4.6 for details. SDs of two to three replicates are shown by error bars.

Hydrolysis of the polymers was evaluated according to the release of glucose and by TLC analysis of reaction products. The commercial enzymes hydrolyzed amylose, amylopectin and glycogen rapidly and with the expected pattern of products (Figure 7). Dextrans were hydrolyzed only by the amyloglycosidase, and the release of glucose was minimal. *Ba*AG2 and *Sc*MAL62 had no activity on dextrans. However, *Ba*AG2 exhibited moderate, but clearly detectable and recordable exo-hydrolysis of amylose, amylopectin and glycogen. The activity was the highest with amylopectin, and the lowest with amylose (Figure 7). From amylopectin (5 g/L), 0.1 g/L of glucose was released by 24 h, and 0.3 g/L by 72 h of the reaction. In the case of *Sc*MAL62, no hydrolysis of amylose was detected, and hydrolysis of glycogen and amylopectin became detectable only by 72 h of the reaction (Figure 7).

#### 2.4.7. The Transglycosylation of Maltose

**Figure 8:**
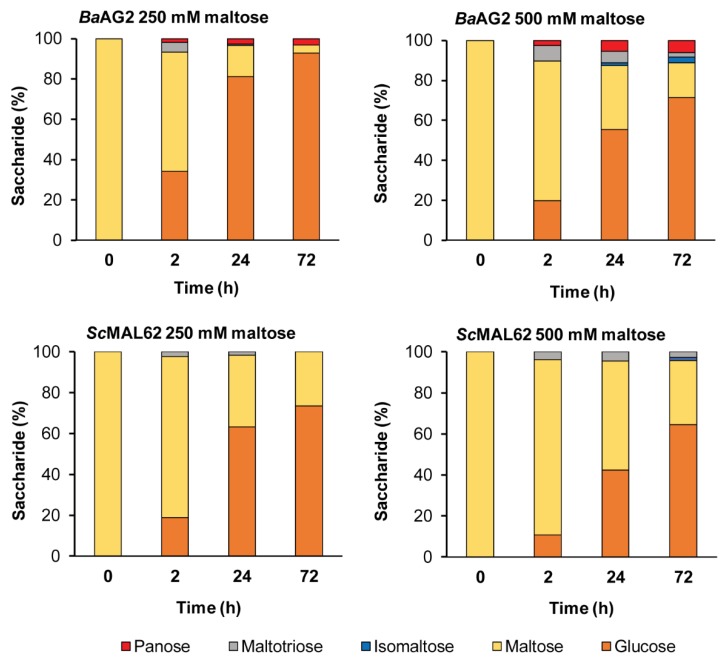
Transglycosylation of maltose by *Ba*AG2 and *Sc*MAL62. 20 µg/mL of the enzyme was reacted with 250 mM or 500 mM of maltose. Samples from the reaction mixtures were withdrawn at designated time points, heated to terminate the reaction and analyzed for sugar composition by HPLC as described in Materials and Methods, paragraphs 4.5. and 4.6. Total amount of detected saccharides at each time point was equalled to 100%. Products were identified using glucose, maltose, isomaltose, maltotriose and panose as references. SDs of two to three HPLC measurements at each time point were up to 20%.

Many α-glucosidases can transglycosylate and produce short oligosaccharides, especially at high concentration of the substrate [17,18,19,20,21]. We assayed this possibility by incubating *Ba*AG2 with 250 and 500 mM maltose up to 72 h and analyzed the reaction products by HPLC. The maltase MAL62 of *S. cerevisiae* was used as a reference. Figure 8 and Table S3 show that already within 2 h at 250 mM (85.6 g/L) maltose concentration, *Ba*AG2 produced maltotriose (4.2 g/L) and panose, α-d-Glc-(1→6)-α-d-Glc-(1→4)-d-Glc (1.6 g/L), in addition to a maltose hydrolysis product—glucose. By 72 h of reaction, the maltotriose content was decreased and panose content increased to 2.6 g/L (Figure 8, Table S3). The *Sc*MAL62 produced only maltotriose under the same conditions, and its amount was considerably lower than in the case of *Ba*AG2—only 2.0 g/L produced by 2 h of reaction (Figure 8, Table S3). Transglycosylation of maltose was enhanced at 500 mM (171.2 g/L) concentration: up to 13.3 g/L of maltotriose was produced by 2 h and 10.4 g/L of panose by 72 h of reaction. Notably, a new transglycosylation product, isomaltose, emerged. It was produced by both enzymes, but its concentration was certainly higher in the case of *Ba*AG2—its concentration in the 72-h reaction sample was 5.2 g/L (Table S3). By 24 h of reaction with 500 mM maltose, the amount of transglycosylation products was 12.6% of total sugars in the reaction mixture, while the respective value for the MAL62 protein was about three times less—4.5% (see Table S3).

We tested panose hydrolysis by *Ba*AG2 and conclude that it accumulated in transglycosylation reaction since it was not hydrolyzed by the enzyme even during extended (22 h) reaction time (Figure S3). In contrast, maltotriose was hydrolysed by *Ba*AG2 (Figure 5, Table 1), and due to that, its content decreased at prolonged transglycosylation reaction (Figure 8, Table S3).

## 3. Discussion

**Figure 9:**
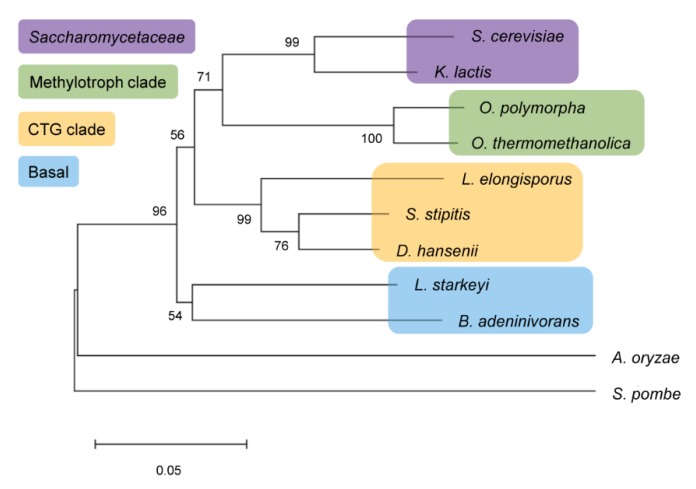
Phylogram based on analysis of D1/D2 domains of large subunit ribosomal RNA (rRNA) gene sequences of ten yeast species and of a filamentous fungus *Aspergillus oryzae*. The bootstrap values (1000 replicates) are shown at the nodes. The scale bar shows the number of base substitutions per site. The Saccharomycotina clades according to [50] are designated by background coloring.

Utilization of α-glucosidic sugars by maltases and isomaltases has earlier been thoroughly studied in *S. cerevisiae* because metabolism of these sugars is crucial in brewing and baking [22,48]. However, transport and intracellular hydrolysis of α-glucosidic sugars have also been investigated in *Ogataea polymorpha* [25] and *Schizosaccharomyces pombe* [33,49]. A maltase has been characterized from *C. albicans* [41] and four maltase-isomaltases from *Scheffersomyces stipitis* [12]. Genes potentially encoding for either maltases, isomaltases or maltase-isomaltases were recently discovered in the genomes of many non-conventional yeasts [12]. Yeast species with deep phylogeny were expected to possess ancient-like α-glucosidases [12]. Figure 9 shows the phylogram of selected yeast species and *A. oryzae* based on sequence analysis of D1/D2 domains of large subunit ribosomal RNA to illustrate the evolutionary relationships between the species. *B. adeninivorans* and *Lipomyces starkeyi* belong to basal group of Saccharomycotina [50]. Based on phylogenetic analysis of orthologous proteins [4], this group diverged from *S. cerevisiae* lineage 200 to 400 million, and from the CTG clade 200 million years ago. The basal group is considered very heterogeneous, its most studied member is *Yarrowia lipolytica* (not presented in Figure 9) and the most basal lineage to this group and all Saccharomycotina is *Lipomyces starkeyi* [50].

On the phylogram (Figure 9), *B. adeninivorans* clusters with *Lipomyces starkeyi*. Genes for eight intracellular α-glucosidases (five maltases and three isomaltases) were predicted in the genome of *L. starkeyi.* In the phylogram of α-glucosidases from non-conventional yeasts, all eight *Lipomyces* enzymes clustered with those of *B. adeninivorans* [12]. However, these putative α-glucosidases of *L. starkeyi* have not been cloned for protein expression and characterization. In Kelly et al. an extracellular α-glucosidase from *L. starkeyi* was biochemically characterized [51]. Unfortunately, the sequence data of this protein is not available. The above-mentioned enzyme of *L. starkeyi* had equally high activity on maltose and isomaltose, but it also hydrolyzed maltotriose, isomaltotriose, panose, amylopectin and starch. Its activity with starch and amylopectin comprised 31 and 42% of that with maltose and isomaltose, and due to this feature, the authors considered it more similar to fungal rather than yeast enzymes [51]. Interestingly, this enzyme did not hydrolyze sucrose and had a quite low activity on *p*NPG. Typically, yeast α-glucosidases hydrolyze *p*NPG more rapidly than maltose. or sucrose [16,24] (Table 1) whereas the opposite is true for maltases of bacteria and archaea [52,53,54].

Our study showed that *Ba*AG2 is a maltase. *Ba*AG2 efficiently hydrolyzed maltose and maltose-like sugars, but could not hydrolyze isomaltose, palatinose nor α-MG that are specific substrates for yeast isomaltases (Figure 5, Table 1). Isomaltose, isomaltose-like sugars as well as acarbose and glucose competitively inhibited *p*NPG hydrolysis by *Ba*AG2 and increased thermostability of the enzyme (Table 2; Figure 6). We also discovered a very strong inhibition of *Ba*AG2 by Tris with the K_i_ of 70.5 μM (Table 2). Tris also considerably increased thermostability of *Ba*AG2 in a DSF assay (Figure 6). As thermostability of *S. cerevisiae* isomaltases was also elevated in the presence of Tris [55], it may bind to the active site of these enzymes. Literature mining revealed Tris as a competitive inhibitor of *Bacillus brevis* maltase with the K_i_ of 14.5 mM [56]. In a yeast *Brettanomyces bruxellensis* (former name *Br. lambicus*)*,* both extra- and intracellular maltases were inhibited not only by acarbose (K_i_ values between 28.5 and 57 μM) but also by Tris (K_i_ values between 7.45 and 15.7 mM) [57]. Compared to *Br. bruxellensis* and *Bacillus brevis* maltases, *Ba*AG2 was much more sensitive to Tris. The pH optimum of *Ba*AG2 was in a moderate acidic region as in the case of other yeast maltases [23,24]. The temperature optimum for *Ba*AG2 was between 40–50 °C, with maximum activity (530 U/mg) achieved at 45 °C. Thermostability of the enzyme was not high—after keeping the enzyme at 45 °C for 30 min, the enzyme’s activity dropped to 46% from the initial. Incubation for 30 min at 50 °C totally inactivated the enzyme (Figure 4). According to literature data, thermolability has been reported for some other yeast α-glucosidases. For example, the four isomaltases of *S. cerevisiae* had all low thermostability. Of those, the IMA1 was most stable, and IMA5 the least stable [55].

According to Hasegawa et al., the MalT protein of *A. oryzae* that has 51% of sequence identity to *Ba*AG2 (Table S2) exhibited *p*NPG-hydrolyzing activity, its expression was induced when grown on maltose, and thereby the MalT was defined by the authors as a maltase [36]. To date no additional data on MalT protein is available. The intracellular maltase (MAL1) protein of *Schizosaccharomyces pombe*, with 43.2% of sequence identity to *Ba*AG2 hydrolyzed *p*NPG and maltose, but had also activity on soluble starch, and some activity on sucrose [33].

Intriguingly, *Ba*AG2 had a detectable hydrolytic activity on MOS with DP up to 7, glycogen, amylose and amylopectin (Figure 7 and Table S4). We assume that the ability to hydrolyze MOS and to cleave polymeric α-glucans, at least to some extent, may be characteristic to maltases of early-diverged yeasts. As *Ba*AG2 is an intracellular enzyme, and this yeast possesses a secreted glucoamylase [9], the maltase *Ba*AG2 has most probably no role in starch degradation. However, yeasts synthesize glycogen as a reserve polysaccharide [58]. Considering that *Ba*AG2 had a remarkable activity on glycogen, the enzyme may contribute to glycogen catabolism in *B. adeninivorans.* We hypothesize that the ability to degrade malto-oligosaccharides and α-glycosidic polysaccharides may be characteristic for the maltases of yeasts with deep phylogeny. Isolation and study of α-glucosidases of the most basal lineages to Saccharomycotina, for example *Lipomyces starkeyi*, should verify the correctness of this hypothesis.

Several GHs of yeasts and filamentous fungi exhibit transglycosylating activity. For example, α-glucosidase of a yeast *X. dendrorhous* (syn. *Pfaffia rhodozyma*) produced from maltose a large proportion of transglycosylation products with α-1,6 linkage, among which panose was the most abundant [17]. It has been shown that the *S. cerevisiae* maltase also produced panose from maltose, yet transglycosylating activity of the *Saccharomyces* enzyme was more than three times smaller compared to the *X. dendrorhous* enzyme. Both enzymes also synthesized isomaltose and maltotriose, but the latter was rapidly used by the enzymes and was therefore not present among the final products [17]. We showed that maltotriose was produced from maltose also by *Ba*AG2 (Figure 8, Table S3), but as it serves as a substrate for the enzyme (Table 1), it was eventually hydrolyzed. α-Glucosidases of filamentous fungi have been suggested as feasible catalysts for transglycosylation. Thus, an *Aspergillus* enzyme with high transglycosylating activity was reported to produce panose and isomaltose from maltose [18,59,60]. When an *A. nidulans* α-glucosidase with strong transglycosylating activity was reacted with 5 g/L maltose during 6 h, approximately 50% of maltose was converted to transglycosylation products, 60% of which was isomaltose [60]. Notably, in addition to maltotriose and panose, isomaltose was also detected among the transglycosylation products produced from maltose by *Ba*AG2 (Figure 8, Table S3). Isomaltose synthesis from maltose was also confirmed for *Sc*MAL62, even though the content of it was only minimal (Figure 8, Table S3).

## 4. Materials and Methods

### 4.1. Yeast and Bacterial Strains, Cultivation of B. adeninivorans

*Blastobotrys* (*Arxula*) *adeninivorans* LS3 (CBS 8244) was kindly provided by Assoc. Prof. V. Passoth (SLU, Uppsala, Sweden). The yeast strain was grown on solid YPD medium (20 g/L peptone, 20 g/L glucose, 10 g/L yeast extract, 20 g/L agar) at 30 °C 24 h for harvesting the cells for genomic DNA extraction. *E. coli* DH5α (Thermo Fisher Scientific, Waltham, MA, USA) was used for DNA cloning and plasmid production. *E. coli* BL21 (DE3) [61] was used for heterologous expression of *Ba*AG2. The ability of *B. adeninivorans* to grow on sugars was assayed as in [12]. Yeast cells grown overnight on BD Difco YNB medium (Thermo Fisher Scientific, Waltham, MA, USA) without amino acids containing 2 g/L glucose were used as inoculum. The YNB medium was supplemented with 2 g/L of a filter-sterilized sugar (glucose, maltose, maltotriose, isomaltose, maltulose, α-methylglucoside, sucrose, raffinose, melibiose, turanose, palatinose, melezitose or trehalose). The cells were incubated on Greiner 96-well flat-bottom transparent polystyrene microplates (Greiner Bio-One, Frickenhausen, Germany) in 200 µl under agitation for 24 h at 37 °C. Optical density of the culture at 600 nm was measured against inoculated medium without sugar at the beginning and end of the experiment using an Infinite M200 PRO microplate reader (Tecan Group Ltd., Männedorf, Switzerland) equipped with Tecan i-control v. 1.7 software from the same provider. Two independent experiments with two parallel measurements were conducted.

### 4.2. Cloning, Heterologous Expression and Protein Purification

Genomic DNA of *B. adeninivorans* was extracted using PowerMax Soil DNA Isolation Kit (MoBio Laboratories, Carlsbad, CA, USA) and the standard protocol by manufacturer. The oligonucleotide primers Ba20130g_PURICter_Fw (5′ **TAACTTTAAGAAGGAGATATACAT***ATGGTTCTAGGATTTTTCAAAAAG* 3′) and Ba20130g_PURICter_Rev (5′ **GCTATTAATGATGATGATGATGAT***GGATTTCATAGATGACTGCCTCCA* 3′), designed according to the gene sequence of *rna_ARAD1D20130g*, were applied to amplify a 1789 bp fragment that represented the coding sequence of *Ba*AG2 [12]. In the primers, the nucleotides annealing with the pURI3Cter vector [62] are shown in bold and those annealing with the *BaAG2* gene sequence are shown in italics. The ATG start codon and the stop codon are underlined in the primer sequences. Recombinant *Pfu* polymerase (Thermo Fisher Scientific, Waltham, MA, USA) and 2.6 ng per µL of reaction mixture of genomic DNA were used in amplification. The PCR product was cloned into a pJET vector from CloneJET PCR Cloning Kit (Thermo Fisher Scientific, Waltham, MA, USA), yielding pJET-BaAG2. To produce the *Ba*AG2 protein with a C-terminal His_6_-tag, an expression plasmid pURI3-AG2Cter was constructed by cloning the *BaAG2* gene into pURI3-Cter vector similarly as in [63]. Insertion of the *BaAG2* gene into the vector was confirmed by DNA sequencing. The cloning procedure was conducted with recombinant *Pfu* polymerase (Thermo Fisher Scientific, Waltham, MA, USA). DNA Clean & Concentrator-5 Kit (Zymo Research, Irvine, CA, USA) was used for purification and concentration of PCR products. Plasmid DNA was purified with FavorPrep Plasmid Extraction Mini Kit (Favorgen Biotech Corp., Ping-Tung, Taiwan). The *MAL62*-containing plasmid pURI-ScMAL62Cter [12] was used to produce the *S. cerevisiae* maltase protein that was analyzed as a reference.

The pURI3-BaAG2Cter and pURI-ScMAL62Cter containing the *BaAG2* gene or *MAL62* gene, respectively, were electroporated into *E. coli* BL21 (DE3) for heterologous expression. A simplified autoinduction medium as in [64] was used for protein overproduction: the LB-based medium (20 g/L tryptone, 5 g/L yeast extract, 5 g/L NaCl) was supplemented with 25 mM phosphate buffer (Na_2_HPO_4_/KH_2_PO_4_; pH 7.2) and 3 g/L glycerol to which filter-sterilized 0.25 g/L glucose and 1 g/L lactose were added. Medium for transformant selection contained ampicillin (Amp, 150 mg/L) for plasmid preservation. Bacterial cells were grown overnight in LB-Amp medium at 37 °C and diluted 100 times in autoinduction medium. At first, the cultures were incubated for 2 h at 37 °C followed by 20-h incubation at 22 °C. The cells were harvested by centrifugation (2400× *g*, 10 min) at 4 °C and stored at −20 °C until further use. Cells were disrupted by sonication with Ultrasonic Homogenizer (Cole-Parmer Instrument Company, Vernon Hills, IL, USA) in 100 mM K-phosphate buffer (pH 6.5) with the cOmplete, EDTA-free Protease Inhibitor Cocktail (Roche Diagnostics, Mannheim, Germany), and centrifuged 30 min at 2400 × *g* at 4 °C. The resulting supernatants were syringe-filtered (pore size 0.45 μm) and loaded onto an IMAC HisTrap FF column coupled with an ÄKTAprime plus chromatography system (GE Healthcare, Uppsala, Sweden). Further purification steps were conducted as described in [63]. Proteins were quantified by measuring the absorbance at 280 nm. The respective extinction coefficients of *Ba*AG2 [133,855 1/(M × cm)] and *Sc*MAL62 [148,990 1/(M × cm)] were computed at ExPASy Proteomics Server (http://expasy.org). Purified proteins were maintained in 100 mM K-phosphate buffer (pH 6.5) at 4 °C.

### 4.3. Determination of Substrate Specificity, Kinetic Parameters and Inhibition

Hydrolysis of *p*NPG was assayed as in [16,40] according to the release of *p*-nitrophenol. 100 mM K-phosphate buffer (pH 6.5) was used and reactions were conducted at 30 °C if not stated otherwise. The purified enzyme was unstable if diluted therefore 5 g/L bovine serum albumin (BSA) was added to the dilution buffer as a protein stabilizer to retain its full catalytic activity. For preliminary assay of substrate specificity of *Ba*AG2, the enzyme was reacted with 100 mM concentration of various potential substrates: maltose, sucrose, maltotriose, isomaltose, melezitose, maltulose, turanose, palatinose or α-MG. At fixed time points, aliquots were withdrawn, combined with three volumes of 200 mM Tris buffer (pH 8.3) and subsequently heated at 96°C for 5 min to stop the reaction. The content of the released glucose was determined spectrophotometrically as in [16,47]. The activities (µmoles of glucose released per minute per mg of protein; U/mg) were calculated from initial velocities of the reaction. For kinetic analysis, initial rates of *p*-nitrophenol or glucose release from substrates were measured at four to seven concentrations ranging from 0.1–3.0 mM for *p*NPG and 2.5–250 mM for di- and trisaccharides. At least three independent measurements for each substrate and concentration were made. *Ba*AG2 content in reaction mixtures ranged from 0.02 to 3.5 µg/mL. The initial velocity data analysis with enzyme kinetics module of the SigmaPlot (Systat Software, San Jose, CA, USA) yielded kinetic parameters (K_m_, V_max_) for the enzyme. *k*_cat_ and *k*_cat_/K_m_ were calculated from these data. The theoretical M_w_ value of 67,901 Da for the *k*_cat_ calculation was computed in ExPASy Proteomics Server (http://expasy.org).

Inhibition of *Ba*AG2 was studied by incubating a suitable amount of enzyme (0.035–0.13 µg/mL) with 0.2–2.0 mM *p*NPG in the presence of following potential inhibitors: 5 μM acarbose, 0.1 mM Tris, 5 mM palatinose, 10 mM isomaltose, 10 mM glucose, 100 mM α-methylglucoside, 100 mM trehalose or 400 mM fructose. The K_i_ values were calculated using enzyme kinetics module of the SigmaPlot (Systat Software, San Jose, CA, USA).

Differential scanning fluorimetry (DSF) was used to evaluate the thermostability of *Ba*AG2 in the presence and absence of ligands. The concentration of *Ba*AG2 was used with 2 µM and for ligands: 100 mM fructose, 50 mM palatinose, 50 mM glucose, 5 mM Tris and 5 mM acarbose. The reaction was conducted in 50 mM 4-(2-hydroxyethyl)-1-piperazineethanesulfonic acid) (HEPES) buffer (pH 7.0) with 150 mM NaCl. The experiment was based on [16,47,65] with above-mentioned modifications. At least two independent experiments were performed with two technical replicates.

Degradation of glucose polymers, i.e., amylopectin-free amylose from potato, amylopectin from potato, glycogen from oysters and dextrans of M_w_ 20 kDa and 110 kDa, was assayed in K-phosphate buffer (100 mM, pH 6.5) containing 0.2 g/L Na-azide. Polysaccharide concentration in the reaction mixture was 5 g/L, and 20 µg/mL of the enzyme was used. At desired time points (2 h, 24 h, 74/96 h) aliquots were withdrawn and heated to stop the reaction. A negative control containing 20 µg/mL BSA instead of the enzyme was incubated alongside. Concentration of released glucose was measured as described above and 3 µl of the samples were analysed on TLC. Maltase from *S. cerevisiae* (*Sc*MAL62), amyloglycosidase from *Aspergillus niger* (Sigma-Aldrich, Merck, Darmstadt, Germany) and α-amylase from *Aspergillus oryzae* (Sigma-Aldrich, Merck, Darmstadt, Germany) were reacted with tested glucose polymers for comparison.

To assay the hydrolysis of malto-oligosaccharides (DP 4‒7), panose and melezitose by *Ba*AG2 and *Sc*MAL62, 50 mM of the sugar was reacted with 2.6 µg/mL of the enzyme in K-phosphate buffer (100 mM, pH 6.5) containing 0.1 g/L of Na-azide and samples were withdrawn at fixed time points. 20 mM isomelezitose (transglycosylation product from sucrose of *Ogatae polymorpha* MAL1) was also tested as a potential substrate. The reaction samples were analyzed using TLC.

### 4.4. Determination of pH and Temperature Optima, Evaluation of Thermostability

Initial velocity of 1 mM *p*NPG hydrolysis by *Ba*AG2 was measured at 30 °C in BSA-supplemented buffers of varied pH (from 3.8 to 8.5) using McIlvaine’s buffer (Na-phosphate/citrate buffer) [66] and 100 mM K-phosphate buffer to cover respective pH-interval, and data were plotted against the pH to determine the pH optimum. Hydrolysis of 1 mM *p*NPG was measured at varied temperatures from 20 to 65 °C. Initial velocity data were plotted against the temperature to reveal the temperature optimum. For thermostability determination, *Ba*AG2 was incubated in 100 mM K-phosphate buffer (pH 6.5) buffer containing 5 g/L BSA for 30 min at temperatures 10, 20, 30, 37, 45 and 50 °C. After cooling the samples on ice, residual activity of the enzyme was determined according to the hydrolysis of 1 mM *p*NPG at 30 °C. Every temperature point was assayed in triplicate. The activity measured after incubation at 10 °C was taken as 100%.

### 4.5. Study of Transglycosylation

20 µg/mL of the enzyme (*Ba*AG2 or *Sc*MAL62) was incubated in 100 mM K-phosphate buffer (pH 6.5) with 0.2 g/L Na-azide and 250 mM or 500 mM maltose at 30 °C up to 72 h. The samples with *Ba*AG2 also contained 5 g/L BSA. Samples were withdrawn at fixed intervals, heated at 95 °C to stop the reaction and analysed on TLC and by HPLC.

### 4.6. Chromatography of Substrates and Reaction Products

To visualize hydrolysis and polymerization products, the TLC analysis was conducted as in [16] on Silica Gel 60 F_254_ plates with concentrating zone (Merck, Darmstadt, Germany). 0.5 µl of the stopped reaction mixture were spotted onto the plate and sugars were separated with two runs in chloroform:acetic acid:water (6:7:1, v:v:v) [67]. For the analysis of products of polysaccharide degradation, 3 µl of the reaction mixtures were spotted. Sugars were visualized by immersion of the plates in aniline-diphenylamine reagent and subsequent heating of the dried plates at 100 °C [68].

HPLC analysis was performed similarly as in [65]. Glucose and fructose were used to calibrate the column. Fructose, maltose, sucrose, isomaltose, palatinose, turanose, maltotriose, panose and melezitose were used as reference sugars.

### 4.7. Alignment of RNA and Protein Sequences and Construction of the Phylogram

Gene sequences from domains 1 and 2 (D1/D2) of large subunit ribosomal RNA were aligned to build a neighbor-joining phylogenetic tree [69] of yeasts using MEGA v. 7.0 [70]. The maximum composite likelihood model [71] with 1000 bootstrap replicates was applied. Protein sequences were aligned using Clustal Omega [72] to calculate identity values of the proteins.

### 4.8. Extraction of Amino Acid Signature from the Alignments and Visualization of Respective Positions on the three-dimensional (3D) Model of S. cerevisiae Isomaltase IMA1

Protein sequences of *S. cerevisiae* maltase MAL62 (UniProtKB: P07265), *S. cerevisiae* isomaltase IMA1 (UniProtKB: YGR287C), *O. polymorpha* maltase-isomaltase MAL1 (UniProtKB: Q9P8G8) and *B. adeninivorans* AG2 were aligned using Clustal Omega [72] and amino acids corresponding to IMA1 signature positions determining the substrate specificity [12,15] were extracted from the alignment. The *S. cerevisiae* IMA1 structure in complex with isomaltose (PDB: 3AXH) [29] was visualized with PyMol [30] and amino acid signature was designated on the structure.

## 5. Conclusions

According to the literature data, a non-conventional yeast *Blastobotrys* (*Arxula) adeninivorans* belonging to the basal group of Saccharomycotina diverged in the evolution of yeasts hundreds of millions of years before *Saccharomyces* and can be considered as a yeast species with deep phylogeny. The genome of *B. adeninivorans* encodes two putative α-glucosidases. In current work, one of them, *Ba*AG2, was produced in *E. coli* and characterized in detail. *Ba*AG2 was proven to be a maltase—hydrolysing α-1,4 and α-1,3 but not α-1,6 linkages in glucose-containing substrates. Interestingly*, Ba*AG2 was strongly and competitively inhibited not only by acarbose, a diabetes drug and a well-known inhibitor of α-glucosidases, but also by Tris. Importantly, at high maltose concentrations, *Ba*AG2 exhibited transglycosylating ability producing potentially prebiotic di- and trisaccharides: isomaltose, panose and maltotriose. Thus, *Ba*AG2 may have a biotechnological value. In contrast to yeast maltases, *Ba*AG2 showed exo-hydrolytic activity on starch, amylose, amylopectin and glycogen. *S. cerevisiae* maltase MAL62 assayed for comparison had only minimal ability towards these polymers and its transglycosylating activity was much lower.

## Figures and Tables

**Table 1:** K_m_, V_max_, *k*_cat_ and *k*_cat_/K_m_ values of hydrolysis of *p*NPG and sugars by *Ba*AG2.

Substrate *	K_m_ ± SD (mM)	*V_max_* ± SD (μmol/(mg × min))	*k*_cat_ ± SD (1/s)	*k*_cat_/K_m_ (1/(mM × s))
*p*NPG	0.76 ± 0.03	751.3 ± 14.5	850.2 ± 16.4	1106.1
Maltose	25.8 ± 1.6	336.4 ± 5.8	380.7 ± 6.5	14.8
Maltotriose	32.5 ± 3.3	117.9 ± 4.4	133.5 ± 5.0	4.1
Sucrose	35.9 ± 2.7	412.4 ± 8.9	466.8 ± 10.1	13.0
Turanose	45.2 ± 6.0	190.1 ± 10.4	215.1 ± 11.9	4.8
Maltulose	7.8 ± 1.0	52.2 ± 1.8	59.1 ± 2.1	7.6
Melezitose	238.3 ± 51.8	231.0 ± 31.8	261.4 ± 36.1	1.1

* For the structure and linkage type of the substrates, see Figure 5. SD, standard deviation.

**Table 2:** K_i_ values and inhibition mode for *Ba*AG2 inhibitors of *p*NPG hydrolysis.

Inhibitor	K_i_ ± SD (mM)	Inhibition Mode
Palatinose	1.4 ± 0.1	Competitive
Isomaltose	22.7 ± 3.0	Competitive
α-MG	21.8 ± 1.4	Competitive
Acarbose	0.83 ± 0.01*	Competitive
Glucose	0.86 ± 0.05	Competitive
Fructose	36.9 ± 2.4	Competitive
Tris	70.5 ± 4.3*	Competitive

* These values are given in µM. SD, standard deviation.

## Supplementary Materials

Supplementary file can be found at https://www.mdpi.com/1422-0067/21/1/297/s1.

## Abbreviations

*Ba*AG2	α-glucosidase 2 of *Blastobotrys adeninivorans*
BSA	bovine serum albumin
DP	degree of polymerization
DSF	differential scanning fluorimetry
GH	glycoside hydrolase
MOS	malto-oligosaccharides
α-MG	α-methylglucoside
PDB	RCSB Protein Data Bank
*p*NPG	*p*-nitrophenyl-α-d-glucopyranoside
OD	optical density
rRNA	ribosomal RNA
*Sc*MAL62	maltase MAL62 of *Saccharomyces cerevisiae*
SD	standard deviation
TLC	thin layer chromatography
T_m_	melting temperature
Tris	tris(hydroxymethyl)aminomethane
YNB	Yeast Nitrogen Base
