# Supplementary material for: Characterization of a Maltase from an Early-Diverged Non-Conventional Yeast Blastobotrys adeninivorans
Source: Int J Mol Sci. 2019 Dec 31;21(1):297. doi: 10.3390/ijms21010297 (PMC6981392; doi:10.3390/ijms21010297)
Supplement: Supplementary file 1 [file ijms-21-00297-s001.pdf]

Supplementary Materials

Characterization of a Maltase from an Early-Diverged Non-Conventional Yeast *Blastobotrys adeninivorans*

Triinu Visnapuu, Aivar Meldre, Kristina Põšnograjeva, Katrin Viigand, Karin Ernits and Tiina Alamäe*

Department of Genetics, Institute of Molecular and Cell Biology, University of Tartu, Riia 23, 51010 Tartu, Estonia

* Correspondence: tiina@alamae.eu (T.A.)

Table S1. Glycoside hydrolases of *Blastobotrys adeninivorans* from families 31, 13 and 15 annotated in the genome* which are predicted to participate in hydrolysis of α-glucosidic oligo- and polymers. *Ba*AG2 that was studied in this work is marked with the grey background.

Protein ID	Gene name	Protein length (aa)	(Predicted) location	GH family	Best hit(s)
3677	*rna_ARAD1C40348g*	840	Intracellular	GH31	α-Glucosidase of *Sporisorium reilianum*, α-glucosidases of *Ustilago*, *Trichoderma*, *Aspergillus*
1696	*rna_ARAD1B19316g*	900	Extracellular	GH31	Secreted invertase AINV of *A. adeninivorans* [1], α-glucosidases of fungi *Thielavia*, *Pyrenophora*, *Ajellomyces*
4522	*rna_ARAD1D13992g*	660	Intracellular	GH31	Hypothetical GH31 proteins of *Phaeosphaeria*, *Trichoderma*, *Penicillium*, *Aspergillus*
3792	*rna_ARAD1C42966g*	931	Extracellular	GH31	Hypothetical α-glucosidase II proteins of fungi
4790 (AG2)	*rna_ARAD1D20130g*	582	Intracellular	GH13	Maltases of *Aspergillus*, *Penicillium* and *Neurospora*
2540 (AG1)	*rna_ARAD1C14212g*	567	Intracellular	GH13	Maltase Mal1 of *Schizosaccharomyces pombe*
3910	*rna_ARAD1C45672g*	625	Extracellular	GH15	Glucoamylase of *A. adeninivorans* [2], similar to glucoamylases of *Cyberlindnera fabianii* and *Rhizopus oryzae*

* The proteins were searched at MycoCosm webpage (https://mycocosm.jgi.doe.gov/mycocosm/home) using a following scheme: Annotations → Cazymes → Glycoside hydrolases →

Table S2. Identity matrix of six α-glucosidases presented in Figure 2 of the main text. Protein sequences were aligned using Clustal Omega [3] to calculate identity values of the proteins.

	*Ha*AG	*Sp*Mal1	*Gs*AG	*Sc*MAL62	*Ao*MalT	***Ba*AG2**
*Ha*AG	100	34.9	36.1	36.6	33.5	**35.1**
*Sp*Mal1	34.9	100	42.1	39.9	43.9	**42.8**
*Gs*AG	36.1	42.1	100	40.6	44.3	**42.5**
*Sc*MAL62	36.6	39.9	40.6	100	46.0	**43.1**
*Ao*MalT	33.5	43.9	44.3	46.0	100	**51.0**
***Ba*AG2**	**35.1**	**42.8**	**42.5**	**43.1**	**51.0**	100

*Ha*AG, *Halomonas* sp. H11 α-glucosidase; *Sp*Mal1, *Schizosaccharomyces pombe* maltase Mal1; *Gs*AG, *Geobacillus stearothermophilus* exo-α-1,4-glucosidase; *Sc*MAL62, *Saccharomyces cerevisiae* maltase MAL62; *Ao*MalT, *Aspergillus oryzae* maltase MalT; *Ba*AG2, *Blastobotrys adeninivorans* maltase AG2.

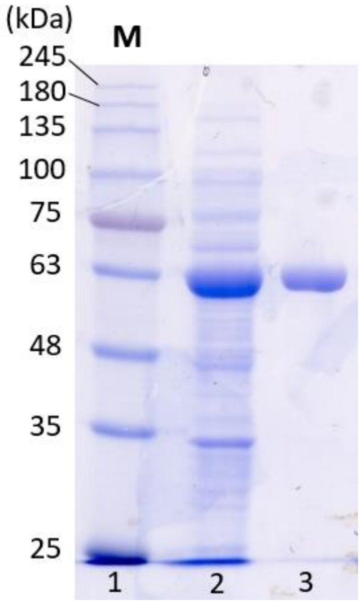

Figure S1. SDS-PAGE analysis of *E. coli* lysate overexpressing *Ba*AG2 (15 µg; lane 2) and purified *Ba*AG2 protein (1 µg; lane 3). Protein Ladder Blue Prestained (Naxo, Tartu, Estonia) was used as a protein size marker (M; lane 1).

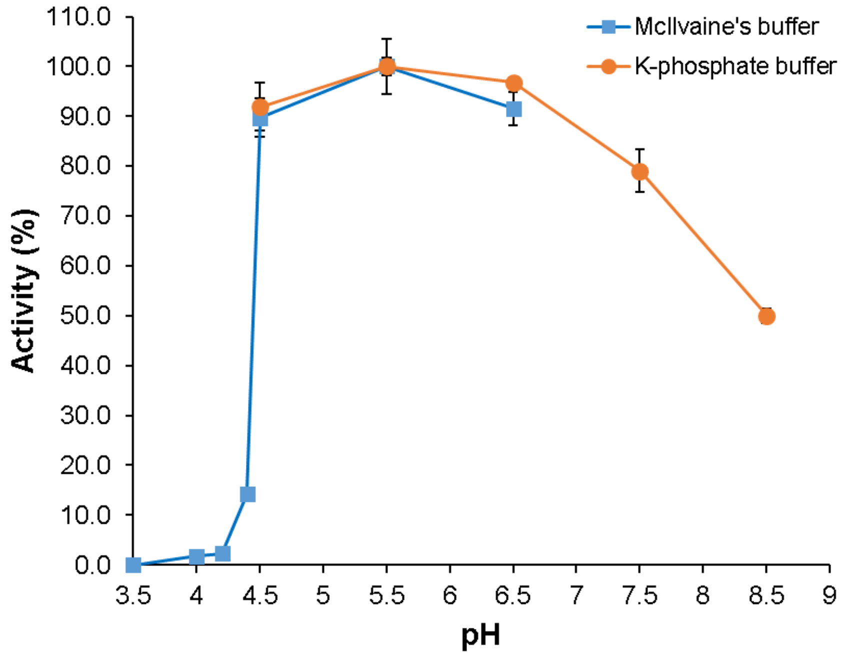

Figure S2. The effect of pH on activity of *Ba*AG2. The activity of the enzyme was determined at 30 °C with 1 mM *p*NPG as a substrate (see Materials and Methods, paragraph 4.4. for details). At each pH value two to three replicates in the same buffer were analysed. Standard deviations between parallels are shown. Activity value 423 ± 7 μmol/(mg×min) was taken as 100% in McIlvaine's buffer (pH 5.5) and 416 ± 23 μmol/(mg×min) as 100% in K-phosphate buffer at pH 5.5.

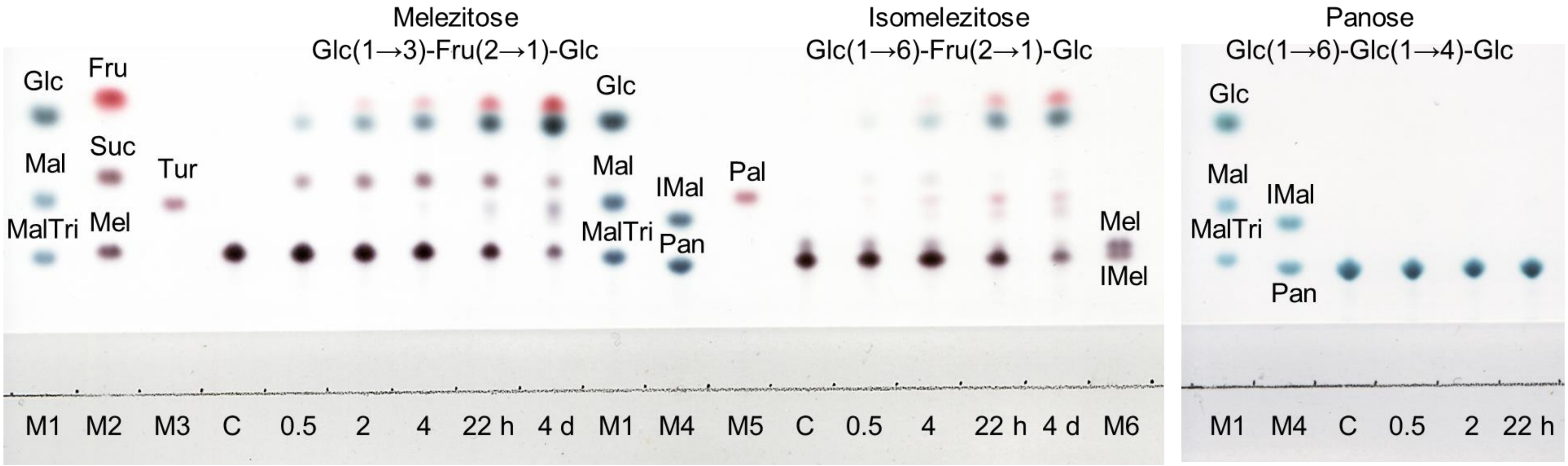

Figure S3. TLC analysis of *Ba*AG2 reaction products from melezitose, isomelezitose and panose. *Ba*AG2 content in the reaction mixtures was 2.6 μg/mL and substrate concentrations 50 mM for melezitose or panose and 20 mM for isomelezitose. Reaction was conducted in 100 mM K-phosphate buffer (pH 6.5) with 5 g/L BSA and 0.2 g/L Na-azide at 30 °C up to 4 days. Samples were withdrawn at indicated time points, stopped by heating and diluted 2 times in milliQ water. 0.5 μl of the mixture was spotted on TLC plates alongside with reference sugars: M1 – Glc, glucose (30 mM) and Mal, maltose (10 mM) and MalTri, maltotriose (10 mM); M2 – Fru, fructose (30 mM) and Suc, sucrose (10 mM) and Mel, melezitose (10 mM); M3 – Tur, turanose (10 mM); M4 – IMal, isomaltose (10 mM) and Pan, panose (10 mM); M5 – Pal, palatinose (10 mM); M6 – Mel, melezitose (5 mM) and IMel, isomelezitose (5 mM). C – control sample without enzyme but with 5 g/L BSA incubated 22 h in the same conditions. TLC plates were developed and visualized as shown in Materials and Methods, paragraph 4.6.

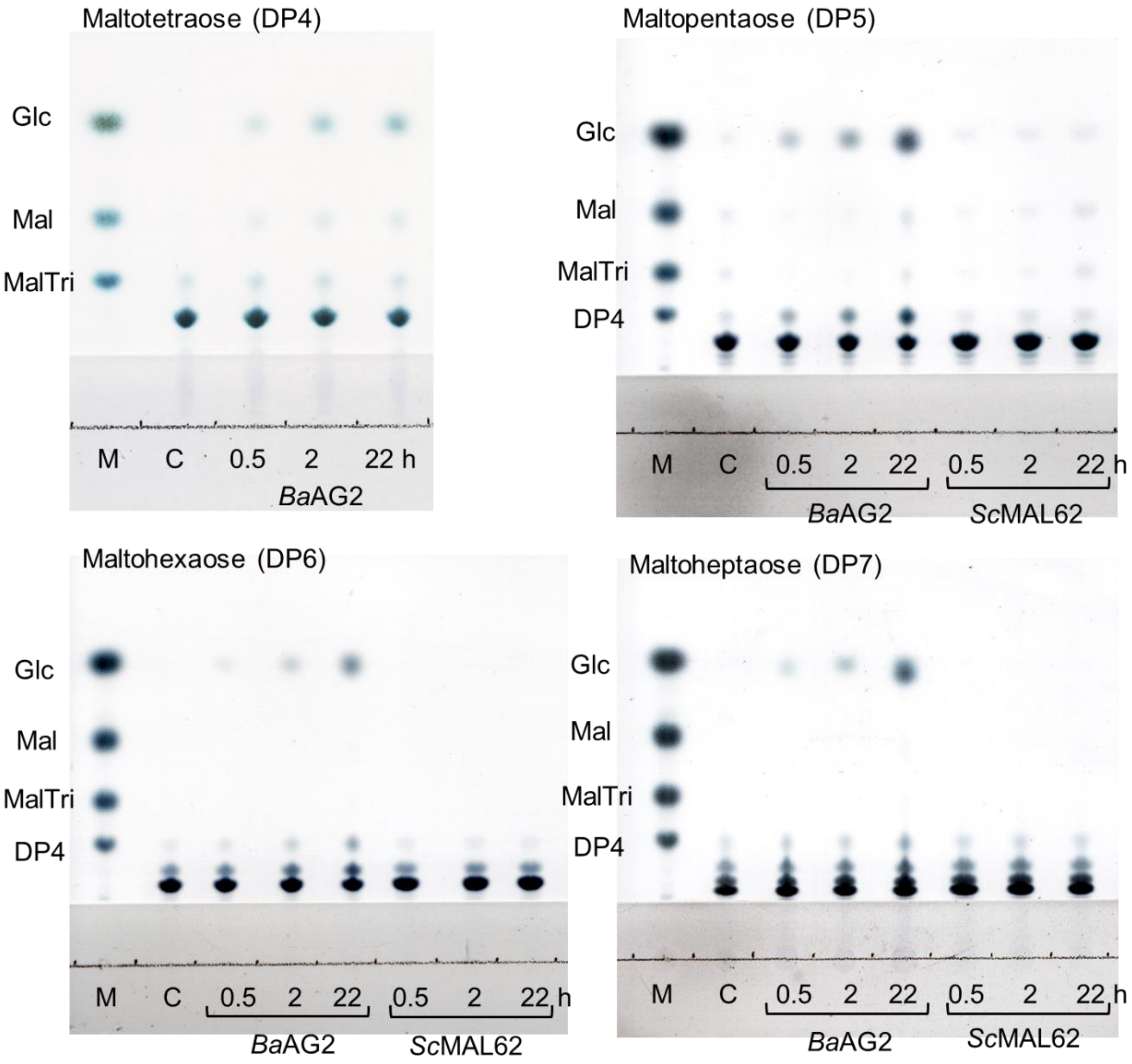

Figure S4. TLC analysis of *Ba*AG2 and *Sc*MAL62 reaction products from maltooliosaccharides DP 4–7. *Ba*AG2 or *Sc*MAL62 content in the reaction mixtures was 3 µg/mL and substrate concentration was 50 mM. The reaction was conducted in 100 mM K-phosphate buffer (pH 6.5; with 5 g/L BSA for the *Ba*AG2 reactions) with 0.2 g/L Na-azide at 30 °C up to 22 h. Samples were withdrawn at indicated time points, stopped by heating and diluted 2 times in milliQ water. 0.5 µl of the mixture was spotted on TLC plates alongside with reference sugars: M – Glc, glucose (30 mM) and Mal, maltose (10 mM) and MalTri, maltotriose (10 mM) and DP4, maltotetraose (10 mM). C – control sample without enzyme but with 5 g/L BSA incubated in the same conditions for 22 h. TLC plates were developed and visualized as shown in Materials and Methods, paragraph 4.6.

Table S3. Transglycosylation of maltose by *Ba*AG2 and *Sc*MAL62 (see Figure 8 of the main text). The enzymes were reacted with 250 mM or 500 mM maltose, samples were withdrawn at designated time points, heated to terminate the reaction and analysed for sugar content. Saccharides and their content in the samples were determined by HPLC as described in Materials and Methods, paragraph 4.6. Average values of two to three HPLC measurements at each time point are shown. Standard deviations were up to 20%.

**Enzyme**	**Substrate**	**Saccharide***	**Time (h)**			
			**0**	**2**	**24**	**72**
			**Saccharide content (g/L)**			
*Ba*AG2	Maltose (0.25 M; 85.6 g/L)	glucose	ND	24.9	70.0	79.4
		maltose	87.4	50.4	13.4	3.5
		isomaltose	ND	ND	ND	ND
		maltotriose	ND	4.2	0.6	ND
		panose	ND	1.6	2.2	2.6
*Ba*AG2	Maltose (0.5 M; 171.2 g/L)	glucose	ND	34.0	98.8	123.2
		maltose	172.5	115.9	56.9	29.9
		isomaltose	ND	ND	2.6	5.2
		maltotriose	ND	13.3	10.0	3.7
		panose	ND	4.4	9.8	10.4
*Sc*Mal62	Maltose (0.25 M; 85.6 g/L)	glucose	ND	16.2	51.8	60.7
		maltose	87.4	62.5	28.7	21.8
		isomaltose	ND	ND	ND	ND
		maltotriose	ND	2.0	1.4	ND
		panose	ND	ND	ND	ND
*Sc*Mal62	Maltose (0.5 M; 171.2 g/L)	glucose	ND	18.6	73.1	114.2
		maltose	172.5	147.7	91.4	55.3
		isomaltose	ND	ND	ND	2.6
		maltotriose	ND	6.6	7.7	5.0
		panose	ND	ND	ND	ND

* Saccharides were identified by HPLC using glucose, maltose, isomaltose, maltotriose and panose as references.
ND, not determined (under detection limit).

References

1. Böer, E.; Wartmann, T.; Luther, B.; Manteuffel, R.; Bode, R.; Gellissen, G.; Kunze, G. Characterization of the *AINV* gene and the encoded invertase from the dimorphic yeast *Arxula adeninivorans*. *Antonie van Leeuwenhoek* **2004**, *86*, 121–134.
2. Bui, D.M.; Kunze, I.; Förster, S.; Wartmann, T.; Horstmann, C.; Manteuffel, R.; Kunze, G. Cloning and expression of an *Arxula adeninivorans* glucoamylase gene in *Saccharomyces cerevisiae*. *Appl. Microbiol. Biotechnol.* **1996**, *44*, 610–619.
3. Sievers, F.; Higgins, D.G. Clustal Omega for making accurate alignments of many protein sequences. *Protein Sci.* **2018**, *27*, 135–145.
